# Reconstruction of the perineal defect after abdominoperineal resection with vertical rectus abdominis myocutaneous (VRAM) flap versus primary direct closure - a single center retrospective cohort study

**DOI:** 10.1007/s00423-025-03804-7

**Published:** 2025-07-09

**Authors:** Georgi Kalev, Sylvia Buettner, Mohamad El-Ahmar, Christoph Reissfelder, Steffen Seyfried, Georgi Vassilev, Julia Hardt

**Affiliations:** 1 Department of Surgery, Medical Faculty Mannheim, University Medical Center Mannheim, Heidelberg University, Theodor-Kutzer-Ufer 1-3, D-68167 Mannheim, Germany; 2 Department of Biometry and Statistics, Medical Faculty Mannheim, University Medical Center Mannheim, Heidelberg University, Theodor-Kutzer-Ufer 1-3, D-68167 Mannheim, Germany

**Keywords:** Abdominoperineal resection, Pelvic surgery, VRAM, Pelvic abscess

## Abstract

**Purpose:**

The closure of the perineal defect following pelvic surgery that includes abdomino-perineal resection (APR) can often be challenging and remains an important issue given the reported high wound morbidity. The vertical rectus abdominis myocutaneous (VRAM) flap, which was proposed in the past as an alternative to direct closure (DC), enables the reconstruction of extensive perineal wounds.

**Methods:**

184 consecutive patients who underwent abdominoperineal resection at tertiary university institution between January 2014 and June 2024 were included in this retrospective analysis. The aim of the study was to evaluate the outcomes of perineal wound reconstruction using a VRAM flap (*n* = 29) compared to DC (*n* = 155).

**Results:**

The rate of overall perineal and abdominal wall wound complications did not differ significantly (*p* = 0.321 and *p* = 1.000, respectively). However, significantly more pelvic abscesses requiring CT-guided percutaneous drainage were observed in the DC-group (*p* = 0.048). Subgroup analysis of patients who underwent radiochemotherapy prior to surgery revealed lower incidence of severe perineal complications after VRAM flap reconstruction of the perineal defect (*p* = 0.037).

**Conclusion:**

The VRAM flap has proven to be a safe technique and should be considered especially for the reconstruction of extensive perineal defects following pelvic surgery.

## Introduction

Abdominoperineal resection (APR) is mainly performed for pelvic malignancies, especially in patients with rectal cancer where the tumor infiltrates the anal sphincter or levator ani muscle, as well as in patients with anal cancer (usually squamous cell carcinoma) that persists or recurs locally after chemoradiotherapy (CRT). The resulting perianal defect can be reconstructed either by direct wound closure (layered suture) or by a pedicle flap. The surgical treatment of locally advanced tumors of the pelvic organs that infiltrate far beyond the organ margins usually involves extended multivisceral resection or pelvic exenteration, leading to a large perineal wound. In this case, direct primary closure of the perineal wound is not always possible due to the large distance between the wound edges, necessitating transposition of a musculocutaneous flap from another body region to facilitate wound closure.

Impaired wound healing is observed in up to 60% [1, 2] of patients following pelvic surgery with direct primary wound closure and poses a serious clinical issue. The high rate of local complications prompted the search for an optimal closure technique for the perineal wound. The results of several studies suggest that the flap reconstruction may be preferable to direct closure, especially in patients who have previously received CRT [3, 4]. Various pedicled, perforated or free flaps have been reported, which can generally be categorized as abdominal-based, thigh-based or perineal-based [3].

The aim of this retrospective study was to investigate the outcomes of vertical rectus abdominis (VRAM) flap reconstruction following pelvic surgery in a tertiary university center and to compare them with outcomes of direct perineal wound closure. The primary objective was to analyze the rate of perianal wound complications - dehiscence, pelvic abscess requiring CT-guided percutaneous drainage (CT-PD), wound complications requiring surgical reintervention, flap necrosis and donor site complications. Secondary objectives included assessment of potential risk factors for impaired wound healing.

## Patients and methods

### Patients

In this retrospective study, we analyzed consecutive patients who underwent APR alone or as part of a multivisceral resection/pelvic exenteration and compared the outcomes of VRAM flap reconstruction with those of direct closure of the resulting perineal defect at a single university center. The data was collected from the in- and outpatient electronic medical records of patients who underwent surgery between January 2014 and June 2024. Included were all patients aged 18 years or older who had received APR/pelvic exenteration at our institution during the specified period, regardless of the underlying diagnosis. Patients who underwent intersphincteric APR or emergency procedures were not considered in the analysis.

APR was performed by a colorectal surgeon (five surgeons were involved over the whole time period) in all patients, with permanent colostomy usually placed on the lower left side of the abdomen. The same surgeon with substantial experience in pelvic surgery performed a multivisceral resection when additional bone or urogenital organ involvement was present. The ileal conduit urinary diversion after cystectomy was carried out by a urologist. In patients who underwent a cystectomy and non-continent urinary diversions, the distal end of the ileal conduit was passed through the right lower abdominal wall, creating an additional stoma (urostomy). In patients with rectal or anal carcinoma the extent of the levator ani resection was adjusted to local situation.

Multivisceral resection was defined as APR combined with an additional resection of at least one other pelvic organ or part of it. Pelvic exenteration involves removing the bladder (urethra), rectum and anus with the sphincter muscle and, in men, additionally the prostate and seminal vesicles and, in women, the vagina, cervix, uterus, fallopian tubes, ovaries and, if necessary, the vulva.

Depending on the size of the defect in the cutaneous plane and the extent of the empty pelvic cavity, a decision was made as to whether the perineal wound should be closed directly by layered suture or using a VRAM flap during the restorative part of the procedure. Indications for VRAM flap reconstruction included large perineal skin defects, typically in recurrent anal cancer, and complete or near-complete levator ani resection. The VRAM flap was also created entirely by the colorectal surgeon. Closure of the donor site was achieved through direct suture in all patients. No mesh augmentation was required. Subcutaneous drains were routinely placed at the perineal site to prevent seroma formation.

### Vertical rectus abdominis musculocutaneous flap (VRAM) - surgical technique

**Fig. 1:**
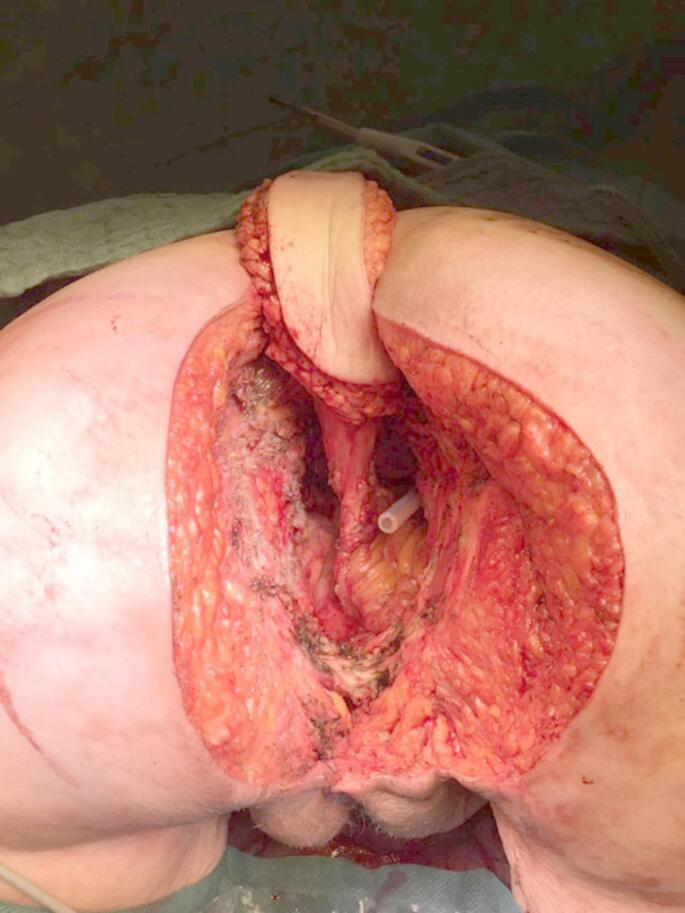
Completely mobilized VRAM flap

**Fig. 2:**
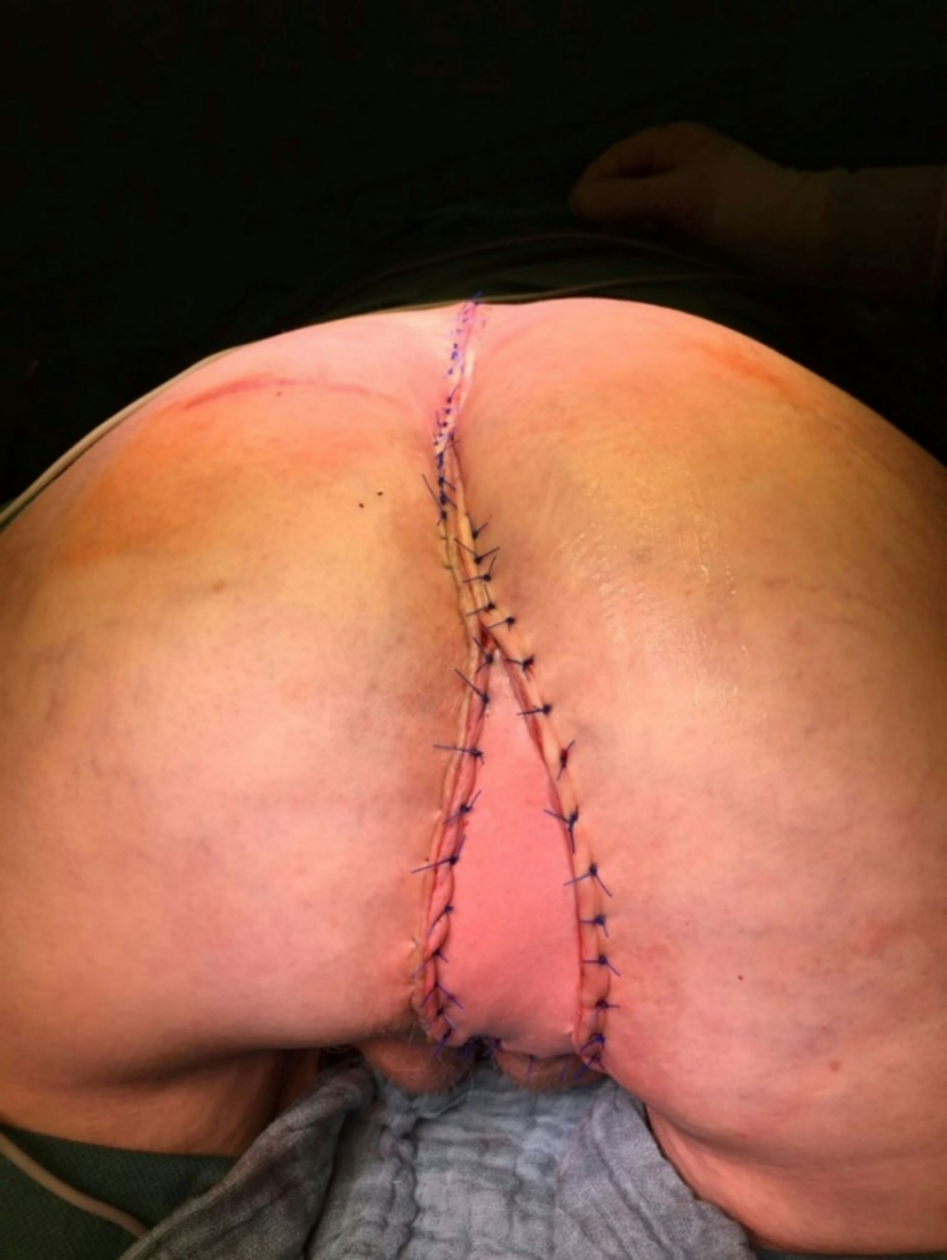
Perineal defect reconstructed using VRAM flap

For perineal wound closure in the flap group, a vertical rectus abdominis myocutaneous (VRAM) flap was used, nourished by the inferior epigastric vessels. Since a definitive colostomy was placed through the left rectus abdominis muscle (RAM) in almost all patients, the right RAM served primarily as the donor site. First, the outline of the RAM and skin island with the required size were marked on the abdominal wall. An incision was then made in the midline from above the umbilicus to the pubis, cutting around the umbilicus on the right side. The subcutaneous tissue in the midline is dissected towards the linea alba, and then the peritoneal cavity is entered. In the next step, rectal resection with TME is carried out in the standard manner. After the surgical specimen has been retrieved, the dissection of the VRAM flap takes place. An elliptical incision is made along the skin marking from the right costal margin (left costal margin for left-sided VRAM flap) to the midline approximately at the level of the umbilicus. The RAM is then dissected from its fascial sheath, whereby the anterior fascial sheath remains attached to the harvested muscle above the arcuate line, and below this line only the muscle is taken (Fig. 1). Attention is paid to the inferior epigastric vessels during the entire dissection, the superior epigastric vessels are ligated. The RAM is then transected at its origin on the costal margin, while the origin at the symphysis remains intact. In the next step, the pedicled musculocutaneous flap is rotated 180 degrees and relocated to the perineal wound. The flap is then sewn into the perineal wound edges in several layers (Fig. 2). Lastly, the anterior abdominal wall is closed.

### Study aims and objectives

The primary objective of the study was to assess the healing of perineal wound. Any deviation from wound healing by primary intention was defined as wound complication and classified as minor if wound dehiscence occurred resulting in healing by secondary intention not requiring further intervention, and as severe if CT-PD or surgical revision of the perineal wound under general anesthesia was required. In addition, we analyzed the rate of flap loss due to flap necrosis as a result of insufficient blood supply.

Disturbances in perineal wound healing were not classified based on the size of the wound dehiscence in the skin plane, as the size of deep wound cavity is a far more relevant determinant that depends on the individual pelvic anatomy of each patient. Another objective was the assessment of the wound complications at the abdominal wall/donor site - ranging from superficial to a full thickness dehiscence (“burst abdomen”). The rate of abdominal wall and perineal hernias was not objective of this study. As not all patients had an underlying oncological disease, these patients did not routinely undergo a CT scan postoperatively as part of the follow-up examinations and therefore there was no objective evidence of whether a hernia occurred or not.

In addition, we assessed the influence of the following factors on wound healing: advanced age (over 75 years), diabetes mellitus, comorbidities (ASA score), severe (< 35 g/l) and mild (< 25 g/l) hypoalbuminemia, radiation, chemotherapy, atherosclerosis, surgery for local recurrence.

### Ethics

Approval for this study was obtained from the local ethics committee at the authors’ affiliated institution with a waiver of informed consent.

### Statistical analysis

Statistical analyses were performed using SAS (Statistical Analysis System) for Windows, Version 9.4 (SAS Institute, Inc., Cary, North Carolina, USA). Absolute and relative frequencies were quoted for qualitative parameters. Mean and standard deviation were calculated for normally distributed quantitative variables, while the median and interquartile range (IQR) are given for skewed parameters. To identify a relationship between two qualitative parameters, the chi-square test or, if necessary due to the data, Fisher’s exact test was used. Shapiro-Wilk test was applied in order to determine the distribution form of quantitative parameters. To compare two groups with regard to a quantitative variable, the t-test was used for normally distributed data and the Mann-Whitney U-test for skewed data. A significance level of *p* = 0.05 was defined.

## Results

During the study period, a total of 184 consecutive patients who underwent APR at the Department of Surgery, University Medical Center Mannheim, Mannheim, Germany, met the inclusion criteria and were included in the analysis. The perineal wound was reconstructed using a VRAM flap in 29 patients (VRAM-group) and through direct closure in the remaining 155 patients (DC-group). A gluteal flap augmented the perineal reconstruction with a VRAM flap in one patient (VRAM group) and the direct closure in another patient (DC group).

Multivisceral resection was performed in 14 (48%) patients from the VRAM-group with 3 (10%) patients undergoing total pelvic exenteration. In the DC-group, in 56 (36%) patients multivisceral resection was carried out, of which 28 (18%) underwent total pelvic exenteration.

The baseline demographics and clinical characteristics of the study population are presented in Table 1.

**Table 1:** Baseline characteristics of all 184 included patients

	VRAM-group n = 29	DC^a^-group n = 155	*p*-value
Age^b^ (years)	66 (IQR 17)	67 (IQR17)	*p* = 0.192
Sex (female/male)	15/14	67/88	*p* = 0.398
Diagnosis (n)			***p*** ** = 0.002**
Rectal cancer	13 (45%)	93 (60%)	
SCCA^c^	12 (41%)	18 (12%)	**p = 0.0003**
IBD^d^	0	7 (4%)	
Sarcoma	2 (7%)	5 (3%)	
other^e^	2 (7%)	32 (21%)	
malignancy (n)	29 (100%)	136 (88%)	***p*** ** = 0.047**
nCT^f^	23 (79%)	81 (54%)	***p*** ** = 0.013**
scRT^g^	1 (3%)	11 (7%)	*p* = 0.7
ltCRT^h^	22 (76%)	66 (43%)	***p*** ** = 0.001**
omentoplasty (n)	21 (72%)	95 (61%)	*p* = 0.255
length of stay^b^ (days)	24 (IQR 17)	14 (IQR 14)	***p*** ** = 0.001**
ASA score (n)			*p* = 0.116
I	8 (28%)	23 (15%)	
II	10 (34%)	85 (55%)	
III	11 (38%)	42 (27%)	
IV	0	5 (3%)	
Laparoscopic/robotic surgery	0	50 (32%)	
Surgery for recurrent tumor^i^ (n)	3 (10.3%)	27 (19.9%)	*p* = 0.228
Mulitvisceral resection (n)	14 (48%)	56 (36%)	*p* = 0.226
Cystectomy	4 (14%)	36 (23%)	
Sacrectomy	1 (3%)	11 (7%)	
Vaginectomy^j^	9 (60%)	18 (27%)	
Vulvectomy	3 (20%)	5 (8%)	
Prostatectomy	4 (29%)	18 (21%)	
Diabetes mellitus (n)	3 (10%)	18 (12%)	*p* = 1.000
Atherosclerosis^k^ (n)	4 (14%)	22 (14%)	*p* = 1.000
Albumin^b^ (g/l)	34.7 (IQR 9.85)	34.3 (IQR 7.8)	*p* = 0.636
Follow-up^b^ (months)	30.5 (IQR 48)	25 (IQR 33)	*p* = 0.496

^a^Direct closure

^b^median (interquartile range)

^c^Anal squamous cell carcinoma

^d^Inflammatory bowel disease

^e^melanoma, vulvar cancer, neuroendocrine tumors, squamous cell carcinoma of the vagina etc.

^f^neoadjuvant chemotherapy – CT – 5-FU, FOLFOX, Mitomycin & 5-FU, FOLFIRI, Carboplatin, etc.

^g^short course radiotherapy (25 Gy in 5 fractions)

^h^long-term chemoradiotherapy > 25 Gy

^i^surgery for recurrent cancer after resection of the primary tumor in the past

^j^with or without concomitant hysterectomy

^k^peripheral, coronary & carotid artery disease

A statistically significant difference was found between the two groups in terms of length of stay, with 26/29 patients from the VRAM-group remaining hospitalized for longer than 14 days after the initial surgery (median length of stay in the DC-group was 14 days). A detailed analysis of the reasons for this finding showed that only 7 patients in the VRAM-group experienced complications related to the perineal wound that led to a prolonged length of stay - surgical revision or comprehensive inpatient wound management. The remaining 19 stayed more than 14 days in hospital, either due to complications unrelated to the peritoneal wound - postoperative delirium, inguinal lymphocele after lymphadenectomy, decompensated liver cirrhosis, postoperative paralytic ileus, necessary repeat surgery after initial R1 resection etc. or due to difficulties arranging home care.

All 30 patients (in both groups) with histologically confirmed anal carcinoma (SCCA) underwent surgery after local failure of the definitive RT or CRT. One patient with SCCA (DC-group) underwent a re-operation because of a local recurrence following definitive CRT and surgery. Among the patients with rectal cancer, four DC patients underwent surgery for local failure after a watch-and-wait strategy following neoadjuvant CRT. One patient with rectal cancer in the VRAM-group and 21 patients in the DC-group underwent repeat surgery for local recurrence after resection, with two patients from the DC-group being operated on after having multiple resections in the past. Considering all malignancies, three patients (10.3%) in the VRAM-group and 27 patients (19.9%) in the DC-group underwent repeat surgery after local recurrence (*p* = 0.228).

Whenever technically possible, an omentoplasty was used – in 95 patients (61.3%) from the DC-group and in 21 patients (72.4%) additionally to the VRAM flap (*p* = 0.255). In each group (VRAM and DC), patients with omentoplasties did not differ significantly from those without pedicled omentoplasty in terms of severe perineal wound complications (*p* = 0.300 and *p* = 0.227, respectively).

### Wound complications/local morbidity

Overall, perineal wound complications (wound dehiscence, CT drainage and/or repeat surgery) were observed in 85 patients (54.8%) in the DC-group compared to 13 patients (44.8%) in the VRAM-group (*p* = 0.321).

The incidence of wound dehiscence that could be conservatively managed was comparable (*p* = 0.741) in both groups – 34.2% (53/155) in the DC-group versus 31% (9/29) in the VRAM-group. A similar proportion of patients in each group returned to the operating room due to perineal wound complications – 14.8% (23/155) DC-group vs. 13.8% (4/29) VRAM-group (*p* = 1.000). However, while in 21/155 (13.5%) patients in the control group a CT-PD was placed due to a pelvic abscess, none of the patients in the VRAM-group required CT-PD (*p* = 0.048). None of the patients in the VRAM-group experienced flap necrosis.

In the DC-group 15 patients developed complications at the abdominal wall surgical site after midline laparotomy. In 14 cases, superficial wound dehiscence occurred at the subcutaneous plane and the wound healed by secondary intention, whereas one patient returned unplanned to the operating room due to a “burst abdomen”. In the VRAM-group, a total of two midline abdominal wound complications (donor site) were observed - in both cases a superficial wound dehiscence occurred. None of the patients experienced a “burst abdomen”. No significant difference was found with respect to superficial (*p* = 1.000), deep (*p* = 1.000), and overall (*p* = 1.000) wound complications.

Table 2 summarizes the analysis of risk factors for severe perineal wound complications in the DC-group. In patients who underwent RT prior to surgery as well as in patients with severe concomitant systemic diseases (ASA score > 2), a significantly higher overall severe local morbidity requiring CT-PD or unplanned repeat surgery was observed. Since none of the patients in the VRAM-group received a CT-PD and only four patients underwent repeat surgery, a reasonable analysis of risk factors for local complications in this group was not feasible.

**Table 2:** Analysis of risk factors for perineal wound complications in the DC-group

	CT-guided percutaneous drainage		Repeat surgery		Overall^а^	
	Odds Ratio (95% Confidence Interval (CI))	*p*-value	Odds Ratio (95% Confidence Interval (CI))	*p*-value	Odds Ratio (95% Confidence Interval (CI))	*p*-value
RT^b^ past^c^ vs. current^d^ > 25 Gy vs. 25 Gy	2.84 (0.79–10.15) 2.42 (0.87–6.75)^1^ 1.19 (1.07–1.32)	*p* = 0.097 *p* = 0.086 *p* = 0.593	3.22 (0.91–11.43) 0.87 (0.32–2.4) 0.48 (0.11-2-11)	*p* = 0.058 *p* = 0.789 *p* = 0.384	3.29 (1.28–8.47) 1.54 (0.68–3.47) 0.93 (0.22–3.89)	***p*** **= 0.011** *p* = 0.301 *p* = 1.000
Intraoperative RT	1.03 (0.12–9.04)	*p* = 1.000	0.92 (0.11–8.31)	*p* = 1.000	1.04 (0.19–5.58)	*p* = 1.000
Diabetes mellitus	2.02 (0.59–6.84)	*p* = 0.272	1.77 (0.53–5.96)	*p* = 0.311	1.85 (0.67–5.16)	*p* = 0.263
Atherosclerosis	1.01 (0.27–3.76)	*p* = 1.000	2.56 (0.88–7.44)	*p* = 0.101	1.66 (0.64–4.31)	*p* = 0.291
nCT^f^	0.82 (0.32–2.10)	*p* = 0.674	1.25 (0.50–3.14)	*p* = 0.629	1.04 (0.50–2.15)	*p* = 0.925
Albumin < 35 g/l	1.67 (0.63–4.40)	*p* = 0.295	1.56 (0.62–3.94)	*p* = 0.341	1.59 (0.77–3.3)	*p* = 0.212
Albumin < 25 g/l	1.62 (0.49–5.39)	*p* = 0.491	0.95 (0.26–3.53)	*p* = 1.000	1.41 (0.53–3.79)	*p* = 0.489
ASA score > 2	1.45 (0.56–3.76)	*p* = 0.447	3.60 (1.45–8.96)	***p*** **= 0.004**	2.76 (1.32–5.79)	***p*** **= 0.006**
Age > 75 years	1.36 (0.51–3.6)	*p* = 0.543	0.53 (0.17–1.68)	*p* = 0.275	0.93 (0.42–2.09)	*p* = 0.869
surgery for local recurrence	1.68 (0.54–5.2)	*p* = 0.354	1.79 (0.62–5.16)	*p* = 0.370	1.79 (0.73–4.37)	*p* = 0.200

^a^CT-guided percutaneous drainage and repeat surgery

^b^radiotherapy

^c^radiotherapy that was completed more than 365 days before surgery

^d^radiotherapy that was completed within the last 365 days prior to surgery

^e^peripheral, coronary & carotid artery disease

^f^neoadjuvant chemotherapy

Subgroup analysis of patients who underwent RT prior to surgery revealed significantly lower incidence of severe perineal complications after VRAM flap compared to direct closure of the perineal defect - Odds Ratio 0.28; 95% Confidence Interval (CI) 0.08–0.99; *p* = 0.037.

## Discussion

Due to the constantly evolving neoadjuvant treatment regimens, APR is nowadays rarely performed as a primary procedure for low and ultra-deep rectal cancer. In patients with SCCA, APR is usually carried out as a salvage procedure for local disease failure occurring with an incidence up to 40% after definitive nCRT or even higher after nRT alone [5, 6]. Extensive salvage resections pose a challenge for the reconstruction of the perineal wound. One of the most studied alternatives to primary direct closure is the reconstruction of the perineal defect using a VRAM flap.

Surgery for malignances is associated with complex perineal wound conditions (previous CT and RT) and larger defects compared to surgery for non-malignant pathologies and therefore may require more often plastic reconstruction. This explains why in our cohort significantly more patients with underlying oncological diseases and thus significantly more patients receiving chemotherapy were found in the VRAM-group. The two groups in our study differed statistically significantly regarding the main diagnosis, whereby the considerably higher proportion of SCCA in the VRAM-group was mainly accountable for this finding. This is not unexpected, as the resulting perineal defect following resection for anal carcinoma is usually much larger than after resection for other oncological entities such as rectal carcinoma.

In our retrospective analysis, a similar incidence of perineal wound dehiscence was observed in both groups (*p* = 0.741). With 34.2% in the DC-group and 31% in the VRAM-group, the incidence was comparable to the reported incidence in the literature [7–9]. We recorded all types of dehiscence, even the most minor ones where patients dress the wound themselves after discharge and which therefore have no clinical relevance. However, we have decided not to grade wound dehiscence according to its size, as the size of the wound does not have a significant influence on the further course unless an intervention/surgery is required. While no substantial difference was found between the two groups in terms of the number of repeat surgeries due to perineal wound complications (*p* = 1.000), significantly more CT-PD for pelvic abscess were placed in the DC-group (*p* = 0.048). As already emphasized by some authors, flap reconstruction leads to a reduction of the empty space in the pelvic cavity [10, 11]. This should reduce the risk of fluid accumulation, which can become contaminated resulting in a presacral abscess. This hypothesis could be confirmed in practice by our retrospective data analysis. Similar results with a significantly lower incidence of perineal abscesses were reported by Butler et al. who studied 111 patients during a 12-year period [7]. In another study, again significantly more patients developed pelvic abscess after direct perineal wound closure − 26.9%, compared to 10.1% in the VRAM-group (*p* < 0.01) [9].

The rate of perineal wound complications mandating surgical revision (14.8% in the DC-group vs. 13.8% in the VRAM-group) was similar or even lower in our study than in other reports. In Butler’s paper, surgical debridement of the perineal wound was performed in 8 patients (11%) in the non-flap group and in three patients (9%) in the group with flaps, also without significant differences between the groups [7]. Chan et al. reported major perineal wound complications requiring reoperation or debridement in three patients (14%) following primary perineal wound closure and five patients (17%) after flap reconstruction [12]. A comprehensive literature search revealed reoperation rates in the primary closure group (up to 25.6%) and in the flap group (up to 44%) that do not differ significantly (OR, 1.05; 95% CI, 0.49 to 2.25; *p* = 0.91) [13]. Studies that included only patients with anal cancer showed even higher rates (up to 30%) of perineal wound infections necessitating surgical revision [14, 15]. A retrospective study found that major (> 2 cm of dehiscence, reoperation required, or readmission) as well as minor (dehiscence < 2 cm, stitch abscesses, or sinus tracts) wound complications were more common in patients with anal cancer than in those with rectal cancer [16]. A plausible explanation could be the more extensive perineal resection in anal cancer and the often more aggressive local radiotherapy. This must be considered when interpreting the outcomes in our cohort, as significantly more patients in the VRAM-group (*p* = 0.0003) had anal carcinoma as an underlying disease.

Regarding the acute complications of abdominal surgical incisions, we found no difference between the two groups. In particular, there was no difference between the occurrence of fascial dehiscence at the donor site in the VRAM-group and at the midline incision in the DC-group. Butler also found no significant difference in the incidence of abdominal wall complications between the flap group (29%) and the non-flap group (33%). The incidence of abdominal skin or fascial dehiscence and delayed complications such as incisional hernias also did not differ substantially between the two groups. Chessin et al. also found no significant difference between RAM flap patients and patients from the control group in terms of complications related to the abdominal wound, including the occurrence of ventral hernias [17]. Two other studies came to similar findings [11, 18]. The lower complication rate associated with the abdominal wall wound/donor site, which was also demonstrated in the current study, confirms the low morbidity of VRAM flap harvest.

Various factors, most of which not related to local complications at the perineal wound or the donor site, were responsible for a longer length of stay in the VRAM-group. Furthermore, a significantly higher proportion of patients with malignant diseases, particularly anal carcinomas, were found in the VRAM-group. The perineal defect following surgery is substantially larger in these patients and is associated with a larger wound area, which explains the intense pain, the delayed mobilization and the prolonged recovery. Therefore, despite the presence of a statistically significant difference, the length of stay was not considered as an outcome parameter.

While the VRAM flap is often performed by plastic surgeons [7, 19–21], in our institution the reconstructive part of the surgery was carried out in all cases by the same visceral surgeon who had previously conducted the resection. Despite this, not a single flap necrosis was observed, confirming that the technique can be successfully performed by experienced visceral surgeons.

A limitation of this study is its retrospective nature, associated with the well-known disadvantages. Although the size of the VRAM-group is comparable to many other studies [7, 11, 17], it is relatively small with considerably fewer patients than the DC-group. Numerous studies investigated and compared direct wound closure with VRAM flap reconstruction in patients with perineal wounds that can be closed alternatively using either technique. In our patient cohort, a VRAM flap was only applied in cases where direct closure was not technically feasible due to the extent of the defect. Although there was a considerable predetermined selection bias and a clearly far more complex wound situation in the VRAM-group, similar outcomes were found in both groups.

Long-term functional outcomes and patient quality of life following perineal reconstruction were not assessed in the present study. These aspects, however, represent important parameters for evaluating the overall clinical benefit of different reconstruction techniques. A future analysis focusing on these functional aspects may provide further insights, particularly regarding the incidence of perineal hernias, the presence of local discomfort or bulging in the perineal region, and other specific functional outcomes. Such data would significantly enhance the understanding of the long-term implications of VRAM flap reconstruction and its impact on patient quality of life.

## Conclusion

Although the VRAM group corresponded to a negative selection and contained significantly more high-risk patients for perineal wound infection, an equivalent rate of wound complications was achieved and there were even fewer pelvic abscesses requiring CT drainage. Therefore, the results of this study strongly support the use of a VRAM, especially in high-risk constellations for complex wound healing disorder.

## Data Availability

The data that support the findings of this study are not openly available due to reasons of sensitivity and are available from the corresponding author upon reasonable request.
